# Mobile App and Digital System for Patients after Myocardial Infarction (afterAMI): Results from a Randomized Trial

**DOI:** 10.3390/jcm12082886

**Published:** 2023-04-15

**Authors:** Bartosz Krzowski, Maria Boszko, Michał Peller, Paulina Hoffman, Natalia Żurawska, Kamila Skoczylas, Gabriela Osak, Łukasz Kołtowski, Marcin Grabowski, Grzegorz Opolski, Paweł Balsam

**Affiliations:** 1st Chair and Department of Cardiology, Medical University of Warsaw, 02-097 Warszawa, Poland

**Keywords:** acute myocardial infarction, cardiac rehabilitation, mobile application, telemedicine, telehealth

## Abstract

Cardiac rehabilitation after acute myocardial infarction is crucial and improves patients’ prognosis. It aims to optimize cardiovascular risk factors’ control. Providing additional support via mobile applications has been previously suggested. However, data from prospective, randomized trials evaluating digital solutions are scarce. In this study, we aimed to evaluate a mobile application—afterAMI—in the clinical setting and to investigate the impact of a digitally-supported model of care in comparison with standard rehabilitation. A total of 100 patients after myocardial infarction were enrolled. Patients were randomized into groups with either a rehabilitation program and access to afterAMI or standard rehabilitation alone. The primary endpoint was rehospitalizations and/or urgent outpatient visits after 6 months. Cardiovascular risk factors’ control was also analyzed. Median age was 61 years; 65% of the participants were male. This study failed to limit the number of primary endpoint events (8% with app vs. 27% without app; *p* = 0.064). However, patients in the interventional group had lower NT-proBNP levels (*p* = 0.0231) and better knowledge regarding cardiovascular disease risk factors (*p* = 0.0009), despite no differences at baseline. This study showcases how a telemedical tool can be used in the clinical setting.

## 1. Introduction

Cardiovascular diseases (CVD) are the leading cause of death worldwide and one of the biggest challenges of contemporary medicine [1]. Novel invasive procedures and tailored pharmacotherapeutic schemes contributed to significant progress in acute myocardial infarction (AMI) management, resulting in reduced mortality [2]. Still, approximately 10% of AMI patients die within a year after hospital discharge [3]. Optimal CVD risk factors’ control is one of the most important components of secondary prevention, which has been highlighted many times [4]. According to the data reported by Jankowski et al., approximately only one in 30 patients after AMI had all CVD controlled according to recommended values [5]. Moreover, target cholesterol levels have been tightened up by now, so the actual percentage of patients who are optimally managed is likely to be even lower. What is more, at least a quarter of patients after AMI in Poland die within 5 years from the event [6]. Despite recent advances in cardiac rehabilitation (CR) programs in Poland [7], only one in three patients undergoes CR during the 12 months after AMI [8]. Therefore, there seems to be a significant field to act in order to improve patients’ prognosis, especially since it is estimated that about half of the deaths from recurrent myocardial infarction are believed to be preventable [4]. Numerous efforts have been undertaken to boost CVD risk factors’ control.

Several clinical trials assessed different approaches with the common goal of improving CVD risk factors’ control in patients after AMI. Robust evidence points towards a noticeable growing interest in telemedical tools with mobile applications leading the field. The number of mobile smartphones is globally increasing [9]. Introducing mobile apps into clinical practice seems inevitable and has been recommended by cardiac societies’ guidelines [10]. Several protocols of studies testing the mobile app utility in cardiac patients have already been published [11,12,13]. Interestingly, many of them report promising results and provide evidence of, i.e., improved blood pressure value controls [14]. Widmer et al. carried out a study, which provided the momentum to further research the field of mobile apps’ utility. It has been demonstrated that complementing a conventional CR program with a smartphone-based program improved CVD risk factor control. What is more, a 40% reduction (*p* < 0.05) in rehospitalizations and emergency visits has been observed [15]. Many studies were underpowered to show positive results, but according to a recent metanalysis, mobile apps positively impact CVD risk factor management [16]. Some of the previously published papers indicated that patients with CVD disease are using mobile apps to learn about the underlying disease and medicines [17].

It should also be pointed out that recently many cardiac societies recommended broader use of telehealth solutions [10,18]. The afterAMI study aimed to assess the impact of a mobile app on the number of rehospitalizations and/or urgent outpatient visits, as well as its influence on CVD risk factor control in post-AMI patients. Early results were previously published. After the 30-day follow-up, patients had significantly lower LDL cholesterol (*p* = 0.0007) and NT-proBNP levels (*p* = 0.0231). No other differences were observed in CVD risk factors’ control [19]. This analysis focuses on the final results after a 6month follow-up.

## 2. Materials and Methods

### 2.1. Study Design

**Figure 1:**
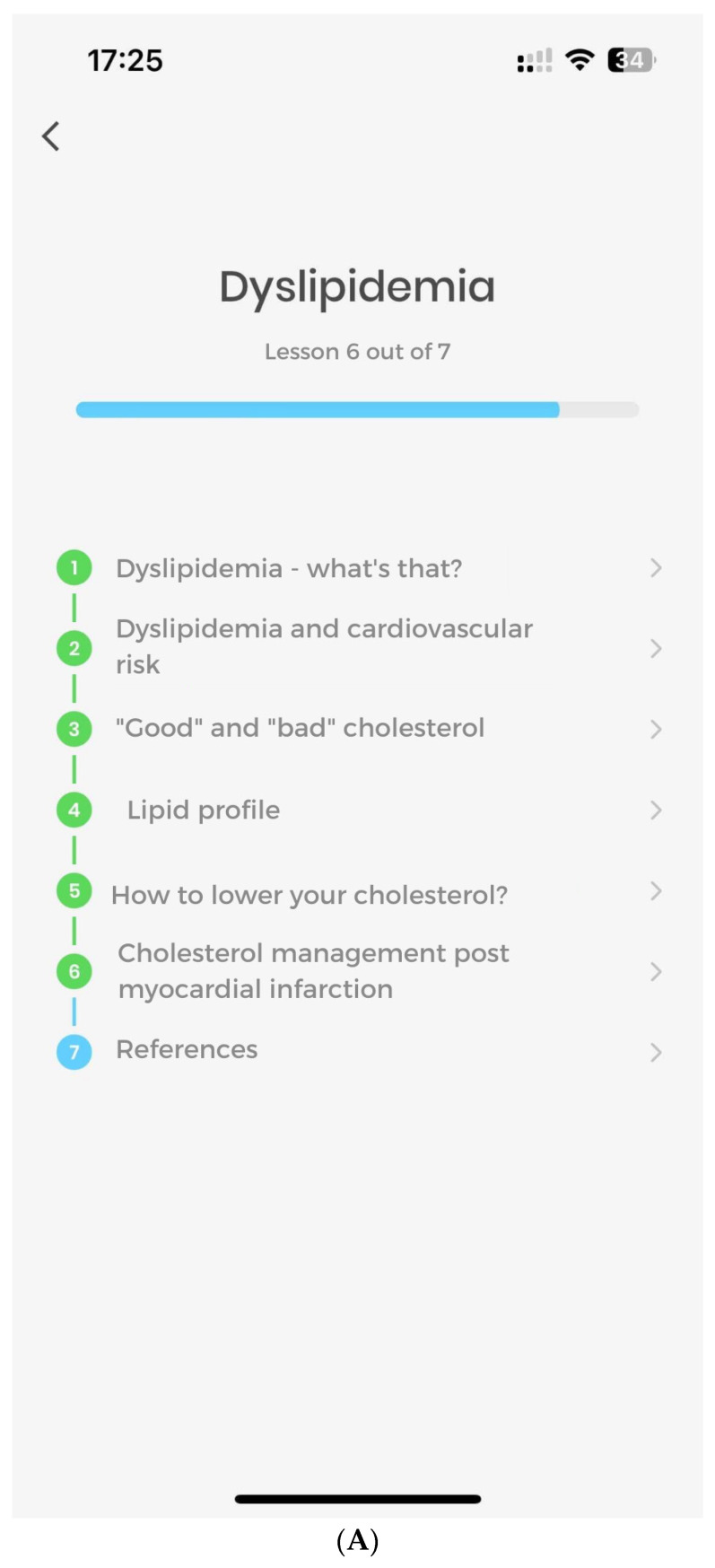

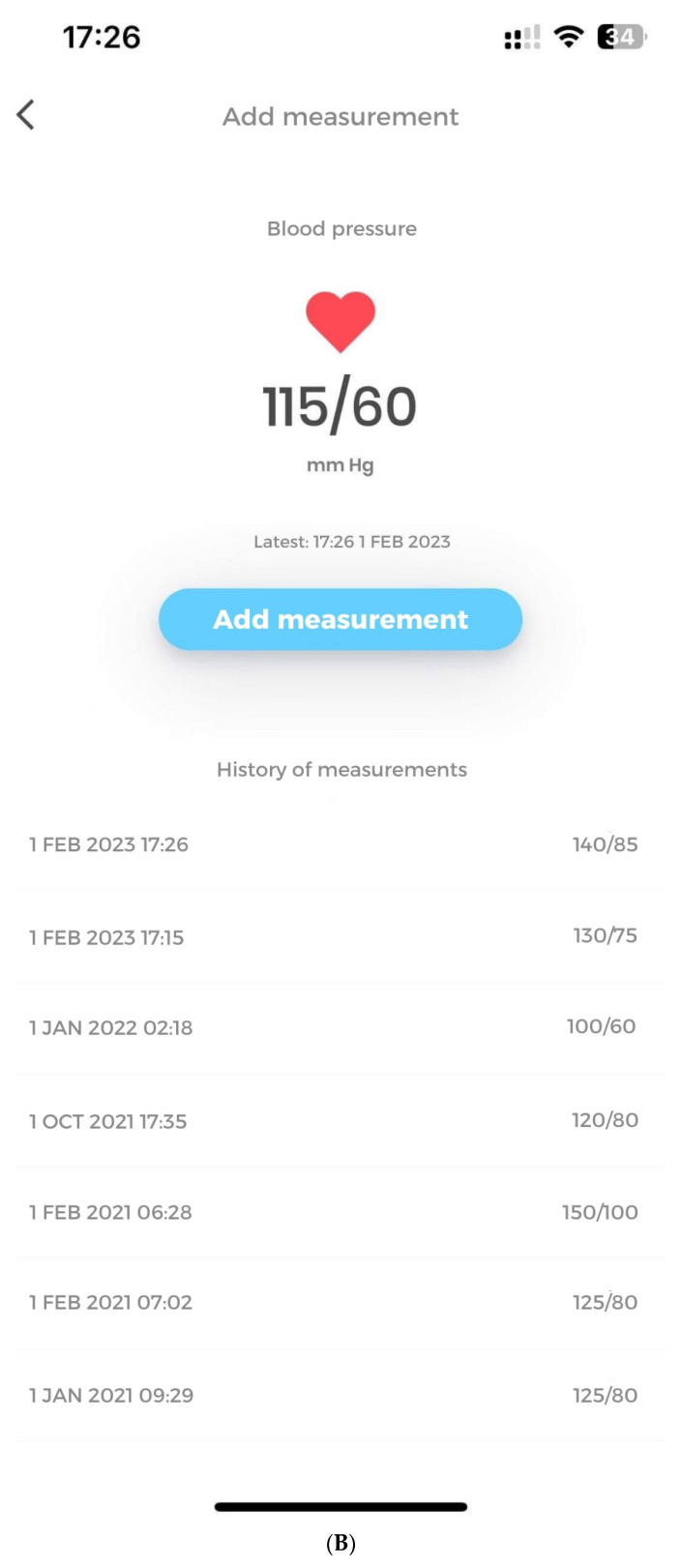

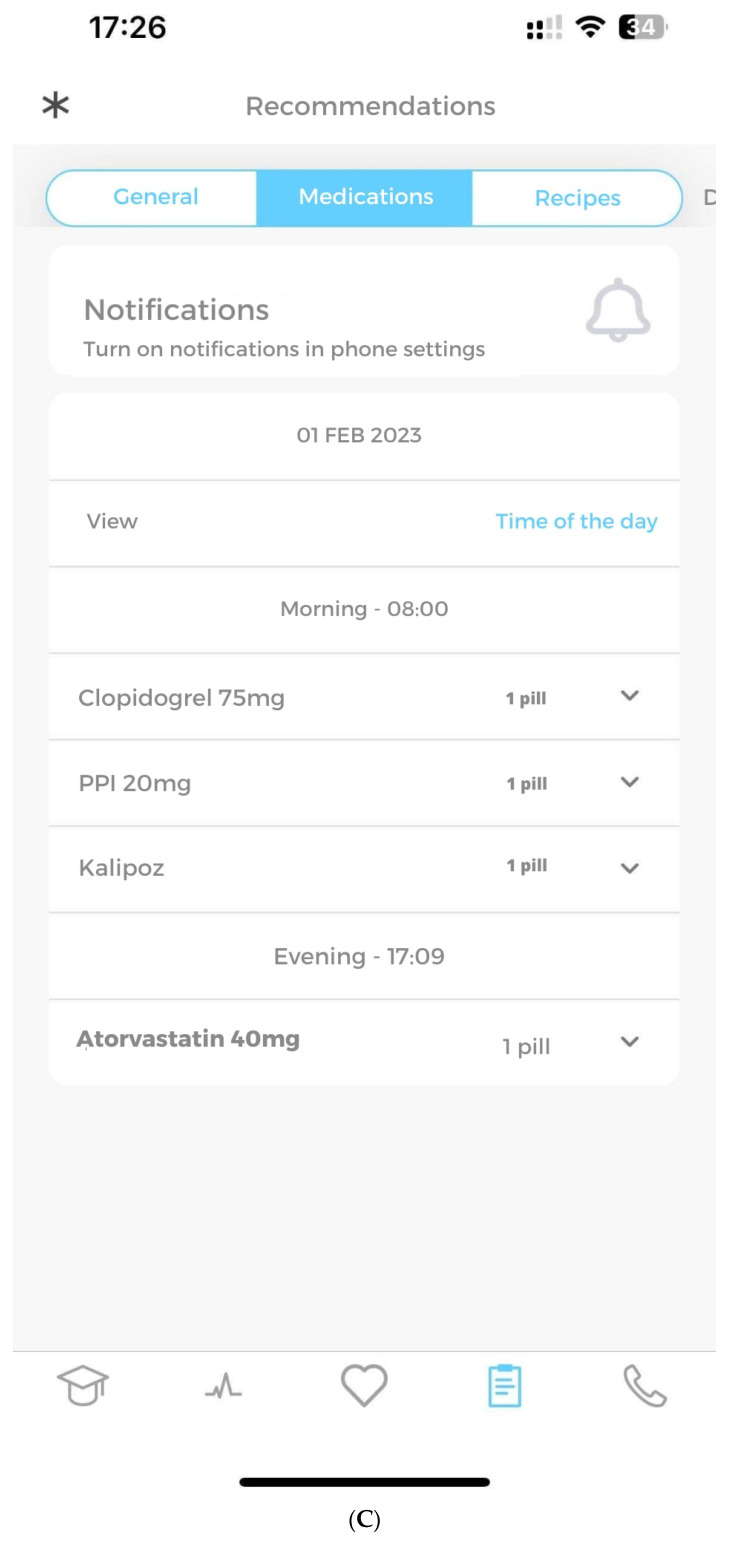
(**A**–**C**) Example screenshots of the afterAMI app.

**Figure 2:**
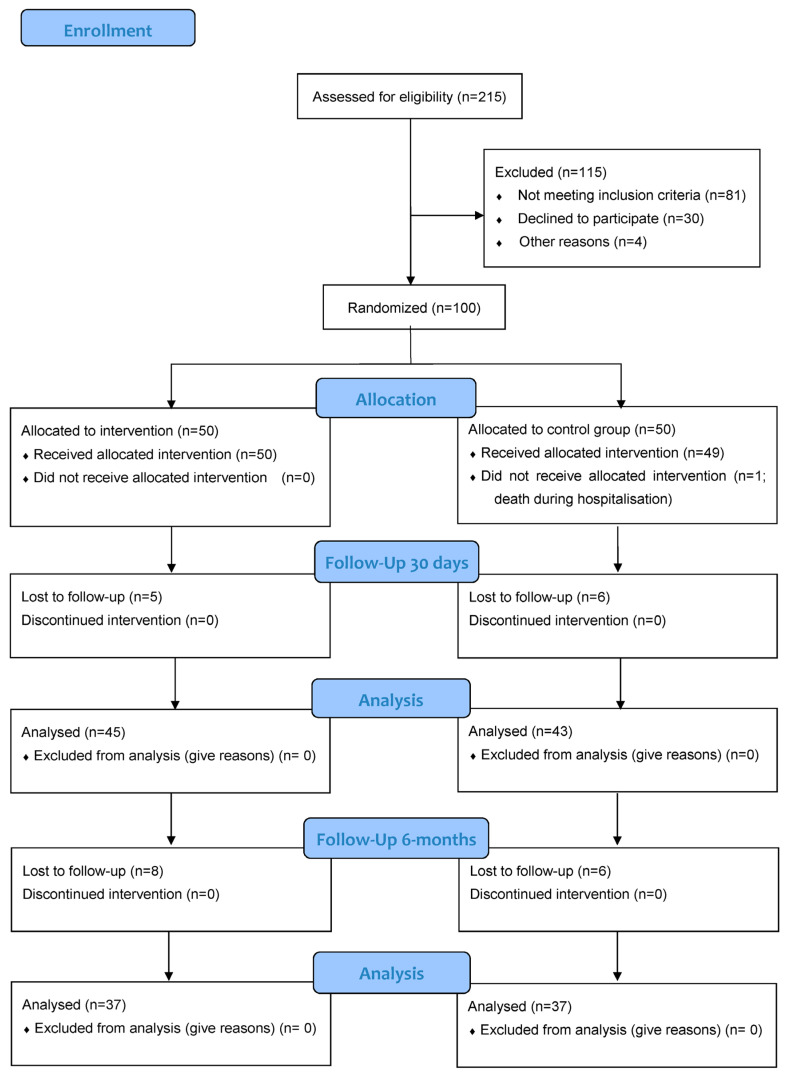
Enrollment and follow-up.

This was a single-center, randomized, open afterAMI trial (mobile app and digital system for patients after myocardial infarction), registered in ClinicalTrials.gov under the number NCT04793425. The study was approved by a local ethical review board (KB/150/2020). The inclusion criteria were as follows: signed informed consent, owning a mobile phone with Internet access and Android/iOS system, hospitalization due to myocardial infarction, age ≥ 18 years old, positive test results (basic mobile applications using skills). Exclusion criteria were: life expectancy < 6 months due to a non-cardiac disease, pregnancy or breastfeeding, negative test results (everyday mobile application use), age < 18 years old, lack of signed informed consent, lack of a mobile phone with Internet access and Android/iOS. Every patient signed informed consent before any study related procedure was conducted. Detailed methods and study design can be found in the study protocol [19]. Briefly, the study involved patients hospitalized due to AMI in a leading cardiac department between 2019–2021. The AMI diagnosis was made based on symptoms, troponin concentrations and ECG results, according to current guidelines [20]. Patients were randomly assigned (1:1) into the intervention group (IG or afterAMI), who received digital support (dedicated mobile app) to standard rehabilitation, or to the control group (CG), which underwent regular cardiac rehabilitation. An independent statistician performed the randomization using a dedicated online tool. The app consists of numerous modules. It provides short articles on a healthy lifestyle, as well as general knowledge on modifiable cardiovascular risk factors. Another feature is short educational messages sent as notifications. The patients can also report their vital signs (e.g., blood pressure, weight, glycemia) and set drug-taking reminders. There is a dedicated module for creating an electronic medical history card, where the patient can note and keep track of all past hospitalizations, underwent procedures, and medical recommendations. Exemplary screenshots from the afterAMI app are presented on Figure 1A–C. Each patient’s account was individually tailored based on the diagnosed comorbidities. Standard rehabilitation consisted of a series of exercise trainings performed on a cycloergometer, as well as dietary and psychological education, and finally follow-up visits. Every patient was provided with extended medical supervision, as all study participants had two additional cardiological consultations. All demographic, clinical, laboratory data, etiology of AMI, as well as drugs at discharge were collected. Endpoints were assessed twice: at 1 month and 6 months after discharge. Please see Figure 2, where a flowchart of the study is presented.

### 2.2. Study Endpoints

The primary outcome was rehospitalization and/or urgent outpatient visit, between baseline and at the 6-month follow-up visit. Secondary outcomes were related to cardiovascular risk factor management: body mass index, blood pressure, dyslipidemia, smoking. The study protocol contains detailed target values of mentioned risk factors [19]. Each value was categorized as being met or not. Other secondary outcomes included cardiovascular risk factor knowledge (CVD risk factors, normal blood pressure values, and recommended lifestyle modifications), as well as return to work. Further data collection covered laboratory test results (including HbA1c and lipid profile) and demographic parameters (sex and age).

### 2.3. Statistical Analysis

The investigator responsible for performing the statistical analysis was blinded. In terms of the endpoints, we looked at the frequency of the events. Regarding secondary endpoints, the change from baseline was assessed. The distribution of continuous variables was estimated using the Shapiro–Wilk test. All continuous variables with a non-normal distribution are presented as median values and interquartile ranges. Continuous variables with a normal distribution are presented as mean values and standard deviations (SD). In the case of variables with normal and non-normal distributions, the groups were compared using Student’s t-test and the non-parametric Mann–Whitney U test. The comparison of qualitative variables between the groups was performed using Fisher’s exact test. For quantitative variables, the change from baseline was assessed. A per-protocol analysis was performed after all of the follow-up visits were completed. We included in the baseline population analysis all patients who met the inclusion criteria and signed an informed consent form, regardless of whether the follow-up was completed. In the case of missing data, the patients were excluded from the particular analysis.

## 3. Results

One hundred patients were enrolled. During hospitalization, 50% (*n* = 50) of them were randomized to the IG and 50% (*n* = 50) to the CG. A total of 25 individuals were lost to follow-up (13 in IG and 11 in CG), and one patient died during the hospitalization, which translated into a 25% attrition rate. One patient did not receive the allocated intervention due to his death during the initial hospitalization, which happened after the consent signing and randomization. This patient was not included in the final results. Patient characteristics can be found in Table 1. The majority of the studied population were male (65%), and the median age of the study group was 61 years. There were some differences between the groups. The individuals assigned to the IG were younger (56.8 ± 9.23 years old vs. 63.42 ± 11.4 in the control group, *p* = 0.0019). Atrial fibrillation and heart failure were more prevalent in the CG.

**Figure 3:**
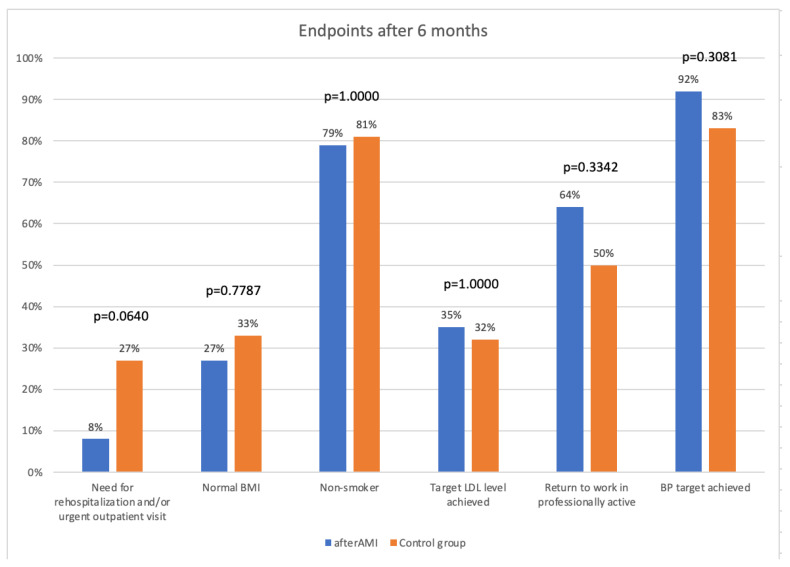
Endpoints 6 months after hospital discharge.

No difference in the rate of the primary endpoint of need for rehospitalization and/or urgent outpatient visit was observed (three [8%] in IG vs. 10 [27%] in CG, *p* = 0.0640). There were no statistically significant differences regarding nicotinism, BMI, meeting LDL target level, meeting target BP, as well as the rate of patients returning to work after AMI. A summary of the results is presented in Figure 3. There was a significant difference regarding knowledge about CVD risk factor observed in favor of the IG (11 points in the test (10–12) vs. nine (8–11) in CG, *p* = 0.0009).

Over the 6-month period, no differences in laboratory results were observed except for NT-proBNP, which was lower in the IG (119 (44–257) in IG vs. 244 (130–696) in CG, *p* = 0.0286), despite no differences observed at the randomization (422 (133–1256) in IG vs. 886.5 (230- 2250) in CG; *p* = 0.0735). The exact laboratory results are presented in Table 2.

## 4. Discussion

The primary finding of this study is that post-AMI patients who receive CR supported by a mobile app do not have significantly lower rates of rehospitalizations and/or urgent outpatient visits. Moreover, there were no differences observed in terms of CVD risk factors’ control. However, patients in the IG had significantly lower NT-proBNP levels when compared with CG, despite no differences at baseline. Additionally, a significant difference regarding CVD risk factor knowledge was observed in favor of patients using the afterAMI app.

AMI is often an important event in the patient’s life, leading to high motivation to improve one’s health status. However, the compliance and adherence decrease over time [21], which makes it difficult to provide CAD patients with continuous care due to common return to previous habits and unhealthy behaviors (sedentary lifestyle, poor diet, smoking). So far, several attempts were made to improve long-term CVD disease risk factors’ management. However, as survival rates within a year after AMI range from 0.94 to 0.68 depending on the age group [6], one could conclude that further improvement is desired. Our study seems to provide valuable evidence into the ongoing search for care optimalization in postinfarction patients.

Participation in CR program is broadly recommended by ESC in patients after AMI [1]. CR extends far beyond just physical exercise and consists of several pillars, among which patient education, psychological support, diet counselling, and CVD risk factor control improvement should be named. Better CVD risk factors’ control contributes to reduced risk of recurrent MI, rehospitalizations, and all-cause mortality, subsequently improving patients’ prognosis [20,21].

Digital solutions have become a subject of extensive research in recent years. Telemedicine can be implemented in numerous forms including: home-based tele-rehabilitation programs, online counseling chatrooms, etc. In recent papers it has been suggested that telehealth CR is associated with similar training intensity and is as cost-effective as conventional outpatient CR [22,23]. What is more, mobile apps have been shown to increase traditional CR completion rates, outcomes, and attendance [24]. Of note, with regard to mobile apps, only a few of those dedicated to CR have been adequately validated. Despite still little evidence, current ESC guidelines on CVD prevention recommend considering mHealth solutions as economically attractive tools, which can contribute to better risk factor control by improving adherence and increasing encouragement in desirable lifestyle modifications [1].

Some of the previous studies on mobile app use in cardiac patients have demonstrated primarily positive effects on selected cardiovascular risk factors, e.g., physical activity [25,26,27]. Conteras et al. showed that hypertensive patients supported by a mobile app had better pharmacological therapeutic adherence resulting in improved BP control [28]. Similarly, in our study, more patients in the IG group met target BP values, but the difference was not statistically significant. Furthermore, lifestyle advice delivered by text messages has been shown to be a useful and cost-effective tool in smoking cessation [29], as well as glycemic control [30]. Mobile apps can also be used for educational purposes. In a recent paper, Min Jung Cho et al. described developing an mHealth solution to be used as a learning instrument dedicated to CAD patients [31]. In our study, patients in the IG who had access to the educational materials had significantly better knowledge of CVD risk factors. Improving health literacy may translate into better adherence and subsequent prognosis in the future.

Considering the results from previous research on mHealth solutions tailored for patients post-AMI, it seems that these novel technologies only contribute to an overall trend towards improved CVD risk factors but often without reaching statistical significance, which is consistent with our findings [14,32]. However, differences in the studied populations, but even more importantly the intervention (mobile app), should be considered. Since each digital intervention is slightly different, comparing obtained results across several trials and finally drawing clear conclusions remains challenging. What is more, lack of differences between studied groups might be a result of an underpowered size of conducted trials.

The reduction in cardiac rehospitalizations is highly desired, as it enables optimizing resource allocation in medical centers. In one of the recently published papers by Indraratna et al., a novel, cost-effective model of care, including a mobile app for patients with heart failure or coronary syndrome, resulted in significant reduction in urgent rehospitalizations and higher rates of completed cardiac rehabilitation [33]. In our analysis, despite showing a trend towards reduction in the primary endpoint event rate, no significance has been shown. However, the population was somehow different, and we also took unplanned outpatient visits into account.

Digitally supported CR is expected to evolve along new, emerging technologies, e.g., wearables and artificial intelligence algorithms [34]. In addition, the barrier of age is highly debated in terms of mobile app implementation. The median age of patients suffering from acute myocardial infarction in Poland is 66.8 years [35], while the mean age of afterAMI study population was 61 years old. Interestingly, AHA recently published a statement on mHealth technologies for cardiovascular disease prevention among elderly patients, which suggests that mobile technology can be effectively used for improving healthy behaviors and medication adherence in this age group [36]. In the statement, it has been stressed that considering the aging of the society, implementing mHealth solutions to improve health outcomes in older adults with CAD is a crucial matter. Therefore, apps such as afterAMI can be regarded as an important move towards the future.

### Limitations

Regardless of the strengths of the study, certain limitations should be considered when analyzing the results. Firstly, the participants in the intervention group were older and had more heart failure and atrial fibrillation, which may be a result of a relatively low number of randomized patients. Additionally, this was a single-center study, and larger studies would provide more information about broader implementation into clinical practice. Secondly, it is worth stressing that not all cardiac patients are capable of using smartphones. In the screened population, every third patient was not able to use a mobile app. Therefore, this solution is not for everyone at this moment. However, we believe that this percentage is expected to grow in the future. It should be stressed that this was not a blinded study because of its nature, which was also stated in other similar studies. Another important limitation that should be mentioned is the high number of patients lost to follow-up. However, one should consider that the study was conducted during the COVID-19 pandemic, which resulted in patients’ fear of infection associated with additional hospital visits and led to their discontinuation of the study. Nevertheless, the investigators were able to collect some of the data via phone calls. As a result, the statistical power of the study has been limited.

## 5. Conclusions

Mobile apps are undoubtedly a field of interest for patients and medical practitioners. The growth of novel tools, programs, and emerging promising results from clinical trials is very encouraging. Telemedicine seems to be currently gaining its momentum. Our study provides new data on mobile app use in AMI patients. A trend towards reduction in rehospitalizations and/or unplanned outpatient visits in AMI patients has been shown. Furthermore, the feasibility of mobile app support in myocardial infarction patients has been proved. The benefits regarding NT-proBNP level may improve long-term prognosis. However, one should consider that mHealth solutions can potentially benefit only selected patients, as some of them are unable to use smartphones. Nevertheless, further research mediated by larger, multicenter studies should be conducted.

## Figures and Tables

**Table 1:** Patient characteristics.

Variable		afterAMI	Control	*p*-Value
**Clinical data**				
Age (years)		56.8 ± 9.23	63.42 ± 11.4	**0.0019**
BMI (kg/m^2^)		28.5 ± 4.06	28.11 ±5.38	0.7247
Body weight (kg)		88.95 ± 13.86	85.47 ± 24.33	0.4625
Sex [1]		34 (68%)	31 (61%)	0.6753
KOS—rehabilitation		17 (34%)	9 (18%)	0.1095
Hospitalization (days)		6 (4–8)	7 (5–11)	0.2143
STEMI		25 (50%)	20 (40%)	0.4176
NSTEMI		25 (50%)	30 (60%)	0.4176
Infarction artery	LAD	26 (52%)	24 (48%)	1
	LCA	15 (30%)	17 (34%)	0.5218
	RCA	16 (32%)	24 (48%)	0.1438
PTCA		39 (78%)	39 (78%)	0.6222
Bypass surgery		5 (10%)	6 (12%)	0.7589
Body weight (kg)		88.9459 ± 13.8663	85.4657 ± 24.3327	0.4625
Height (cm)		176.3 ± 7.2328	171.6 ± 8.9729	**0.0186**
Nicotinism		33 (66%)	32 (64%)	1
Packet years		20 (0–30)	14 (0–32.5)	0.7934
Diabetes, type I		2 (4%)	0 (0%)	0.4949
Diabetes, type II		11 (22%)	11 (22%)	1
Hypertension		30 (60%)	34 (68%)	0.3828
Dyslipidemia		36 (72%)	39 (78%)	0.3069
Atrial fibrillation/atrial flutter		1 (2%)	7 (14%)	**0.0288**
Heart failure		6 (12%)	15 (30%)	**0.0274**
Implanted pacemaker or ICD		1 (2%)	5 (10%)	0.1112
Chronic kidney disease		1 (2%)	1 (2%)	1
Peripheral artery disease		1 (2%)	1 (2%)	1
EF in hospital (%)		51.78 ± 8.42	48.0 ± 9.22	**0.0394**
CVD risk factors knowledge		8 (6–9)	8 (4–9)	0.4131
Employed		27 (54%)	17 (34%)	0.1261
**Lab tests at hospital**				
Troponin I (μg/L)		0.7930 (0.2250–5.5710)	0.694 (0.111–4.350)	0.7248
Troponin II (μg/L)		2.2550 (0.7145–8.7340)	5.640 (0.437–34.635)	0.1702
Creatinine (mg/dL)		0.98 ± 0.21	1.05 ± 0.34	0.1991
eGFR (mL/(min × 1.72 m^2^)))		79.16 ± 17.22	73.28 ± 20.93	0.1351
Na (mmol/L)		139.1 ± 3.05	139.6 ± 4.36	0.5399
K (mmol/L)		4.17 ± 0.45	4.38 ± 0.51	**0.0363**
WBC (×10^9^/L)		10.27 ± 3.04	10.19 ± 2.94	0.9052
HbA1C (%)		5.8 (5.4–7.1)	5.6 (5.4–6.0)	0.4593
NTproBNP (pg/mL)		422 (133–1256)	886.5 (230–2250)	0.0735
HgB (g/dL)		14.58 ± 1.49	14.14 ± 1.83	0.1989
Total cholesterol (mg/dL)		191.3 ± 71. 57	192.1 ± 52.29	0.9523
HDL (mg/dL)		39.55 ± 10.02	46.78 ± 10.65	0.0010
LDL (mg/dL)		117.5 ± 68.59	111.7 ± 61.56	0.6621
Tg (mg/dL)		146 (92–233)	136.5 (87–201)	0.2423
**Drugs at discharge**				
ACEi		42 (84%)	40 (80%)	0.5229
ARB		4 (8%)	2 (4%)	0.2314
ARNI		0 (0%)	0 (0%)	
MRA		9 (18%)	15 (30%)	0.2366
B-blocker		42 (84%)	41 (82%)	0.7398
CCB		20 (40%)	10 (20%)	**0.0257**
Statin		46 (92%)	45 (90%)	1
Ezetimibe		5 (10%)	2 (4%)	0.2673
VKA		0 (0%)	0 (0%)	
NOAC		1 (2%)	2 (4%)	1
ASA		45 (90%)	43 (86%)	1
Clopidogrel		12 (24%)	13 (26%)	1
Prasugrel		2 (4%)	0 (0%)	0.2419
Ticagrelor		28 (56%)	28 (56%)	1
Digoxin		0 (0%)	0 (0%)	

ACEi—angiotensin-converting-enzyme inhibitors, ARB—angiotensin receptor blockers, ARNI—angiotensin receptor neprilysin inhibitor, ASA—acetylsalicylic acid, BMI—body mass index, CVD—cardiovascular disease, EF—ejection fraction, eGFR—estimated glomerular filtration rate, HbA1C—hemoglobin A1c, HDL—high-density lipoprotein, ICD—implantable cardioverter-defibrillator, KOS—rehabilitation—coordinated care program for patients after myocardial infarction; LAD—left anterior descending artery, LCA—left circumflex artery, LDL—low-density lipoprotein, MRA—aldosterone receptor antagonists, CCB—calcium channel blockers, NOAC—novel oral anticoagulants, NSTEMI—non-ST–elevation myocardial infarction, NTproBNP—N-terminal pro–B-type natriuretic peptide, PTCA—percutaneous transluminal coronary angioplasty, RCA—right coronary artery, STEMI—ST-elevation myocardial infarction, Tg—triglycerides, VKA—vitamin K antagonist, WBC—white blood cells.

**Table 2:** Laboratory results after 6 months.

Endpoint	afterAMI	Control Group	*p*-Value
Creatinine (mg/dL)	0.945 (0.84–1.26)	0.95 (0.80–1.01)	0.4510
eGFR (mL/(min × 1.72 m^2^))	78.18 ± 17.11	69.77 ± 20.10	0.0940
HbA1C (%)	5.8 (5.5–7.7)	5.7 (5.6–6.0)	0.7491
NTproBNP (pg/mL)	119 (44–257)	244 (130–696)	**0.0286**
HgB (g/dL)	14.4 (13.3–14.9)	13.85 (13.3–14.6)	0.3587
Total cholesterol (mg/dL)	130 (114–145)	134 (116–153)	0.5112
HDL (mg/dL)	44 (39–54)	46 (41–61)	0.1990
LDL (mg/dL)	58 (45–75)	64.5 (48.5–83.5)	0.3226
Tg (mg/dL)	98 (71–181)	100 (82.5–135)	0.8800
LDL difference vs. baseline	38.4 ± 50.75	49.44 ± 64.51	0.4721

eGFR—estimated glomerular filtration rate, HbA1C—hemoglobin A1c, HDL—high-density lipoprotein, LDL—low-density lipoprotein, NTproBNP—N-terminal pro–B-type natriuretic peptide, Tg—triglycerides.

## Data Availability

The data underlying this article will be shared on reasonable request to the corresponding author.
